# Primary lung cancer samples cultured under microenvironment-mimetic conditions enrich for mesenchymal stem-like cells that promote metastasis

**DOI:** 10.1038/s41598-019-40519-4

**Published:** 2019-03-12

**Authors:** Douglas Saforo, Linda Omer, Andrei Smolenkov, Aditya Barve, Lavona Casson, Nolan Boyd, Geoffrey Clark, Leah Siskind, Levi Beverly

**Affiliations:** 1 University of Louisville, Department of Pharmacology & Toxicology, Louisville, KY 40202 USA; 2 University of Louisville, Department of Biochemistry & Molecular Genetics, Louisville, KY 40202 USA; 3 University of Louisville, Cardiovascular Innovation Institute, Louisville, KY 40202 USA; 4 University of Louisville, James Graham Brown Cancer Center, Louisville, KY 40202 USA; 5 University of Louisville, Department of Physiology, Louisville, KY 40202 USA; 6 University of Louisville, Department of Medicine, Louisville, KY 40202 USA

## Abstract

The tumor microenvironment (TME) is composed of a heterogeneous biological ecosystem of cellular and non-cellular elements including transformed tumor cells, endothelial cells, immune cells, activated fibroblasts or myofibroblasts, stem and progenitor cells, as well as the cytokines and matrix that they produce. The constituents of the TME stroma are multiple and varied, however cancer associated fibroblasts (CAF) and their contribution to the TME are important in tumor progression. CAF are hypothesized to arise from multiple progenitor cell types, including mesenchymal stem cells. Currently, isolation of TME stroma from patients is complicated by issues such as limited availability of biopsy material and cell stress incurred during lengthy adaptation to atmospheric oxygen (20% O2) in cell culture, limiting pre-clinical studies of patient tumor stromal interactions. Here we describe a microenvironment mimetic *in vitro* cell culturing system that incorporates elements of the *in vivo* lung environment, including lung fibroblast derived extracellular matrix and physiological hypoxia (5% O2). Using this system, we easily isolated and rapidly expanded stromal progenitors from patient lung tumor resections without complex sorting methods or growth supplements. These progenitor populations retained expression of pluripotency markers, secreted factors associated with cancer progression, and enhanced tumor cell growth and metastasis. An understanding of the biology of these progenitor cell populations in a TME-like environment may advance our ability to target these cells and limit their effects on promoting cancer metastasis.

## Introduction

The tumor microenvironment consists of a diverse milieu of transformed and non-transformed cells that ultimately coordinate to build and maintain a physical environment that supports tumor growth and potentiates escape and establishment at secondary systemic sites^1^. These constituents act in concert and dynamically regulate a pathological microenvironment that modulates physical characteristics within the tumor such as tissue stiffness, oxygen tension, and metabolite availability^2–4^. As tumors grow, these elements promote the hallmarks of cancer such as sustaining proliferative signaling, evading immune cell death, inducing angiogenesis, and activating invasion and metastasis^5^. Recent evidence implicates an activated tumor stroma as enablers of these processes^6,7^.

The constituents of the non-tumor elements within the stroma are multiple and varied, however the cancer associated fibroblasts (CAF) are thought to be a major contributor to the TME stroma^7^. CAF currently lack specific markers but display characteristics similar to activated fibroblasts such as expression of alpha-smooth muscle actin (*α*-SMA) and vimentin^8^. They act in paracrine fashion by producing potent proliferative cytokines that elicit mitogenic signaling within tumor cells. Furthermore, these CAF also can directly stimulate angiogenesis via secretion of vascular endothelial growth factor (VEGFA) and activate invasion via production of transforming growth factor beta (TGF-*β*)^9,10^. CAF produce a modified extracellular matrix (ECM) which acts as a bioactive mechanical scaffold that can influence tumor cell survival and metastasis, as well as matrix metalloproteinases (MMP) that allow the dynamic regulation of the ECM architecture through degradation of local matrix proteins and activation of latent growth factors within the matrix^11^.

Despite these critical effects on tumor homeostasis and progression, the origin of the CAF remains elusive and may arise from a heterogeneous number of cell types. Evidence has shown that CAF possess many characteristics of mesenchymal stem cells, implicating these cells as a possible origin of the CAF^12^. Unfortunately, pre-clinical studies of the origin of the tumor stroma are hampered by a number of challenges. Primary samples from patient tumor resections are limited and this material is valuable for the diagnosis of the patient. Primary cancer cell lines may be more sought after from this material, and successful transformation of cancer cell lines currently requires repeated xenografts in mice during which time the tumor microenvironment is replaced by mouse stroma^13^. *In-vitro* methods to obtain cell lines from primary tissue resection are hindered by time to cell isolation, and these cells can acquire changes *in-vitro* during the time it takes to passage them in traditional cell culture conditions. During this time progenitor cell types may differentiate, become quiescent, or undergo apoptosis^14^.

Various strategies have been developed to better isolate progenitor cell types. The ECM, which is well known to modulate cell behavior through mechanism of its mechanical stiffness, protein composition, crosslinking, and bioactive components, has also been shown to improve culture of bone marrow mesenchymal stem cells (MSC)^15^. Culture dishes are frequently coated with components of this extracellular matrix to promote the adhesion and differentiation of a variety of cell types. Previously, we and others have shown that cell-derived extracellular matrices (CDM) are replicative of the *in-vivo* environment and influence cancer cell signaling to recapitulate tumorigenic processes *in-vitro*^16–18^. We have shown that fetal human lung fibroblasts produce a CDM that supports lung adenocarcinoma cells to regulate EMT processes and provide survival signaling to promote survival in conditions of serum starvation and hypoxia^16^.

Hypoxia is a critical factor in the tumor progression of solid cancers^19^. Oxygen tension in physical tissues is around 2–8% and plays a role in regulating metabolic homeostasis. Intratumoral oxygen tension can be much lower (<0.1%)^20^. *In-vitro* systems that control oxygen tension have provided proliferative benefits to a number of stromal cell types compared to traditional culture in atmospheric normoxia (20% O_2_)^21^. Culturing at physiological levels of hypoxia has previously been reported to be critical for the cultivation and maintenance of human stem cells^22^.

We hypothesized that these factors, physiological hypoxia and an *in-vivo*-like extracellular matrix, are crucial to maintain tumor stroma, and that incorporating these elements in a microenvironment mimetic *in-vitro* model would improve survival and cultivation of primary cells from small quantities of patient tumor resections. To test this hypothesis, we collected cells from tumor resections of six patients with non-small cell lung carcinoma (NSCLC) and grew them from isolation in different *in-vitro* environmental conditions. Utilizing a combination of cell derived ECM and physiological hypoxia, we were able to rapidly cultivate and massively expand populations of patient tumor associated stromal progenitors. Though this stroma was derived from early, pre-metastatic, treatment naïve NSCLC it exhibited stem-like characteristics, maintained markers of pluripotency, and enhanced tumor cell growth *in-vitro* and metastasis *in-vivo* in a xenograft mouse model compared to normal lung fibroblast cell lines.

## Results

### Microenvironment mimetic culture system characterization

**Figure 1:**
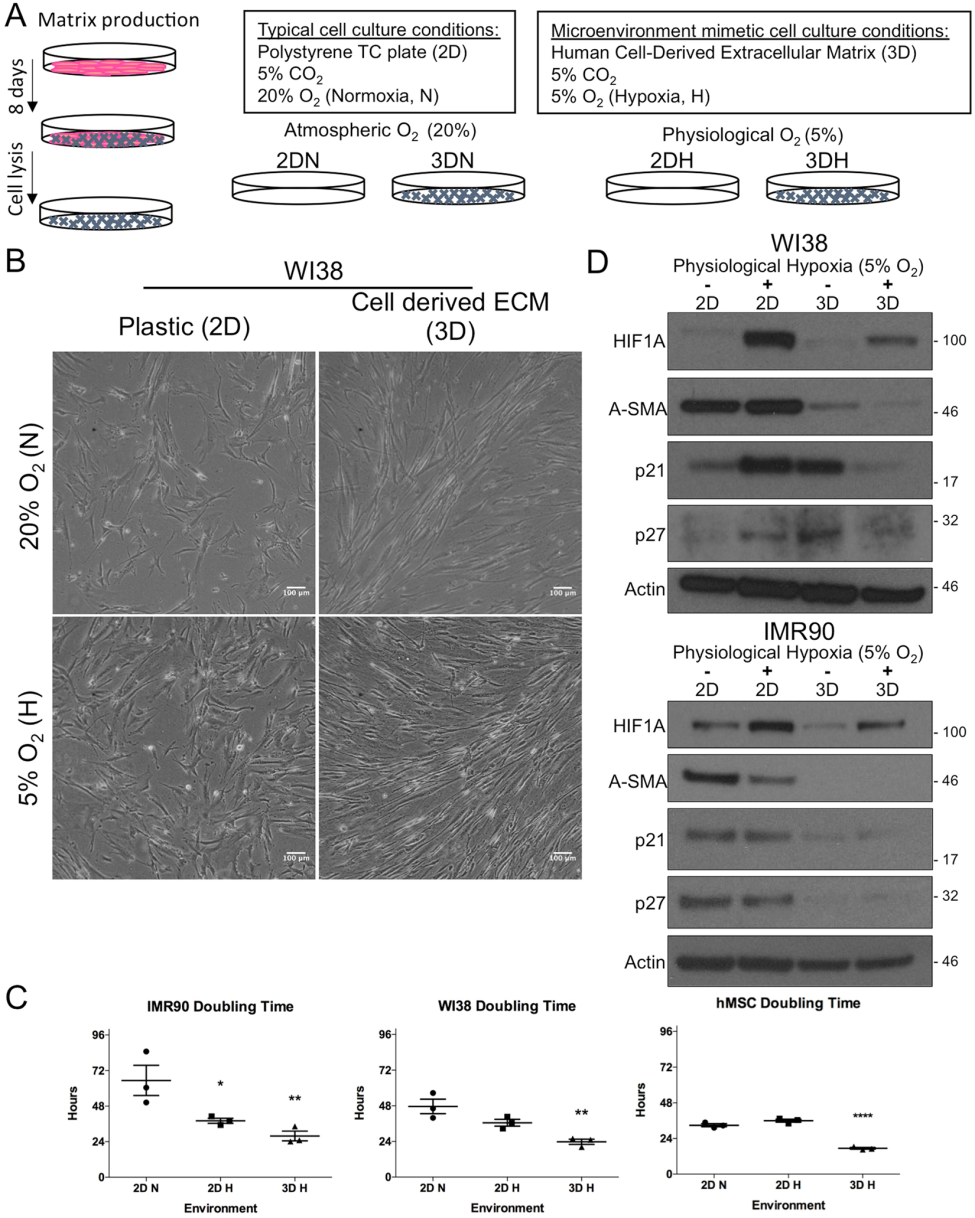
Physiological hypoxia and cell derived matrix enhance proliferation of stromal cell lines and are non-desmoplastic. (**A**) Schematic of cell derived matrix preparation from IMR90 fetal lung fibroblast cell line and characteristics of microenvironment mimetic conditions. (**B**) Phase contrast images of equal numbers WI38 fetal lung fibroblast cells grown for 72 hours in four environmental conditions (2D Normoxia, 2D Hypoxia, 3D Normoxia, and 3D Hypoxia). Scale bars are 100 *μ*m. (**C**) Doubling time of WI38, IMR90, and bone marrow mesenchymal stem cell line hMSC following short term growth in different environments (Statistical analysis performed by ANOVA with Dunnett’s test for multiple comparison to 2DN, *p < 0.05, **p < 0.01, and ****p < 0.0001; data presented as mean ± S.E.M.). (**D**) Western blot analysis of hypoxia inducible factor 1 alpha (Hif1a), alpha smooth muscle actin (*α*-SMA), and cell cycle inhibitors (p21, p27) from WI38 and IMR90 after 72 hours growth in different environments. Cropped images in the figure are derived from full length blots presented in Supplementary Fig. S3.

Various approaches have been used to attempt to isolate progenitor populations from tumors and bone marrow including serum withdrawal and specific conditioned medium, using specialized culture techniques such as hypoxia and extracellular matrix proteins, and culturing cells using 3-dimensional scaffolds or suspension culture. A commonality of these approaches is that each attempt to simulate certain aspects of the physiological condition to limit the growth of non-progenitor cell types and optimize expansion of rare or quiescent progenitors. In order to test the hypothesis that an *in-vitro* culturing system resembling the microenvironment of the human lung would facilitate the isolation and expansion of sensitive primary patient tumor cell populations, we developed a microenvironment mimetic culturing system that includes a fibroblast derived extracellular matrix (ECM) and an atmosphere that maintained the oxygen tension at a physiological level (Fig. 1A).

To characterize this system, we assessed each environmental component individually (tissue culture plastic in atmospheric oxygen (2DN), tissue culture plastic in physiological hypoxia (2DH), cell-derived ECM in atmospheric oxygen (3DN), and cell derived ECM in physiological hypoxia (3DH)), and utilized two fetal human lung fibroblast cell lines, IMR90 and WI38, and a human bone marrow mesenchymal stem cell line (hMSC). After 72 hours of growth in each environment, we observed a qualitative increase in WI38 cell number in single conditions of 2DH or 3DN compared to 2DN, as well as an increase in cell number in 3DH (Fig. 1B). Similarly, we observed the greatest increase in cell number for all cell lines in the microenvironment mimetic system that combined physiological hypoxia and cell-derived extracellular matrix (Fig. S1). Quantification of mean cell doubling time (Fig. 1C) in the 3DH environment showed significant decreases in IMR90 (P < 0.0099), WI38 (P < 0.0039), and hMSC (P < 0.0001), compared to the other conditions. The single condition of physiological hypoxia significantly decreased mean doubling time only for the IMR90 cell line (P < 0.0387).

To assess whether the physiological hypoxia environment induced a measurable oxygen response in the cells, we assessed stabilization of hypoxia-inducible factor 1, a global regulator of oxygen homeostasis. Although western blot analysis revealed a stabilization of hypoxia-inducible factor 1 alpha subunit (HIF1A) in the physiological hypoxia environments (2DH and 3DH) after 72 hours, there was a considerable decrease in HIF1A stabilization in the 3DH environment compared to the 2DH environment (Fig. 1D). Previously, it was reported that cell-derived matrices generated from stroma of various stages of tumor progression can activate fibroblasts to undergo myofibroblastic and desmoplastic activation^8^. To ensure that the matrices from human fetal lung fibroblasts in our system were not inducing a desmoplastic response in the cells, we assessed the expression of alpha smooth muscle actin (A-SMA), a marker of activated myofibroblasts and desmoplastic stroma. We found that A-SMA expression decreased in fibroblasts cultured on cell derived matrix and was variably affected by hypoxia, with a slight decrease in IMR90. Additionally, we investigated regulators of cell cycle progression, and found cell cycle inhibitors p21 Waf1/Cip1 (p21) and p27 Kip1 (p27) were also decreased in the 3DH condition relative to the single 2DH and 3DN conditions. Taken together, our findings suggest that despite long term culture in typical cell culture conditions, commonly used mesenchymal fibroblast and stem cell lines cultured in a microenvironment mimetic system experience a proliferative benefit while not invoking a desmoplastic transformation, properties essential to the establishment of primary cell lines from resected tumor material.

### Enhanced cell isolation and proliferation from patient tumor biopsy

A challenge to preclinical testing of patient tumors is that resected material is limited and precious for obtaining crucial diagnostic information. Progenitor populations within tumors are thought to be a rare subset of this already limited tissue. To test the hypothesis that a microenvironment mimetic cell culture system would improve the yield of sensitive progenitor cells present within tumors, we obtained six primary tumor resections from treatment naïve patients with non-small cell lung carcinoma (NSCLC), including three squamous cell carcinoma and three adenocarcinoma tumors (Table 1). In these patients, metastasis could not be measured at the time of resection (MX) and samples from one of the patients (1072) was later determined to have cancer present in 2 of 11 lymph nodes (N1). Specimens from the remaining tumor resections did not have metastasis present in the lymph nodes. A portion of these resections were prepared by enzymatic digestion to create single cell suspensions, and equal quantities of cells were seeded onto various cell culture conditions.

**Table 1:** Patient sample characteristics.

Specimen Code	Diagnosis	Staging	Histologic Grade	Sex	Race	Age	Cell plating density (cells/cm^2^)
CDSR00**927**	squamous cell	T2aN0MX	III	M	white	56	4300
CDSR00**955**	squamous cell	T1bN0MX	II	M	white	68	Tumor:110000; Normal:12000
CDSR00**956**	adenocarcinoma	T1bN0MX	II	F	black	59	150000
CDSR00**959**	adenocarcinoma	T3N0MX	II	F	white	75	130000
CDSR00**960**	squamous cell	T3N0MX	III	M	white	73	57000
CDSR0**1072**	adenocarcinoma	T2N1MX	III	F	white	75	4444

**Figure 2:**
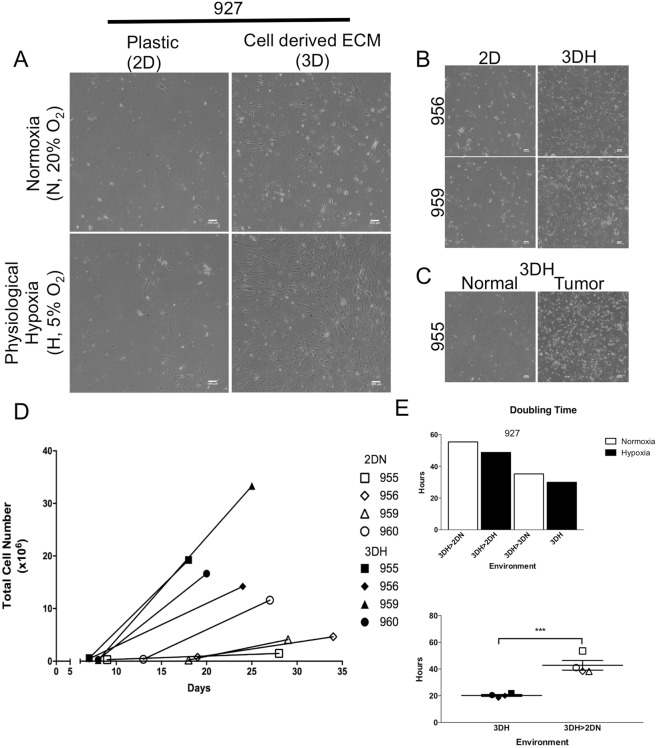
Physiological hypoxia and cell derived matrix synergistically enhance proliferation of Patient Tumor Stromal-like cells. Resected tumors were enzymatically digested with collagenase/dispase and single cell suspensions were grown on four environmental conditions (2DN, 2DH, 3DN, 3DH). (**A**) Representative phase contrast images (from 5 random fields) of patient sample 927 cell growth 7 days post isolation in each condition. Scale bars are 100 *μ*m. (**B**) Phase contrast images of patient samples 956 and 959 obtained 7 and 6 days after isolation in 2DN and 3DH. (**C**) Phase contrast images of patient sample 955 tumor and adjacent normal lung cells 9 days post isolation. (**D**) Total cell numbers and days post isolation of 4 patient samples grown in 2D Normoxia and 3D Hypoxia at the time of first and third passaging. (**E**) (Upper) Doubling time of 3DH derived 927 cell line upon transfer to 4 environmental conditions. (Lower) Doubling time of patient derived cell lines maintained in 3DH or transferred to 2DN. Statistical analysis performed by two-tailed T-test, ***p < 0.001, n = 4; data presented as mean ± S.E.M.

The first sample tested, 927, was seeded onto the four environmental conditions detailed above and monitored with microscopy. Seven days post isolation, we observed cell populations of elongated and spindle cell morphology in every environmental condition (Fig. 2A). There appeared to be an increase in the number of attached cells in the 2DH and 3DN environments compared to the traditional 2DN environment, however, remarkably, we observed massive outgrowths of colonies and migrated cells in the 3DH environment. Cells continued to proliferate in both physiological hypoxia environments but did not passage in the normoxia environments despite presence of extracellular matrix. We found that all tumor samples tested in the 3DH environment showed a marked increase in attached and proliferating cells post isolation compared to traditional cell culture methods (Fig. 2B,D), however we did not observe any increase in primary cell survival from adjacent normal tissue (Fig. 2C). While the 3DH environment greatly enhanced the proliferative capacity of each tumor sample, allowing the first passage after 7 days, in most cases cells grown in traditional methods produced a viable cell line after a moderate delay (Fig. 2D). Similarly, when cells that were isolated in 3DH were transferred to the other environments, we found that the cells were still viable though their proliferation slowed (Fig. 2E). In four biological replicates, we found that the mean doubling time significantly decreased (P < 0.009) in the 3DH environment (20.25 ± 0.7147 h, n = 4) compared to transfer to a traditional cell culture environment (42.82 ± 3.643 h, n = 4). These data indicate that a microenvironment mimetic system indeed accelerates the establishment of a heterogeneous cell line from NSCLC resections compared to traditional culture systems.

### Primary tumor cell mesenchymal lineage characterization and immunophenotyping

**Figure 3:**
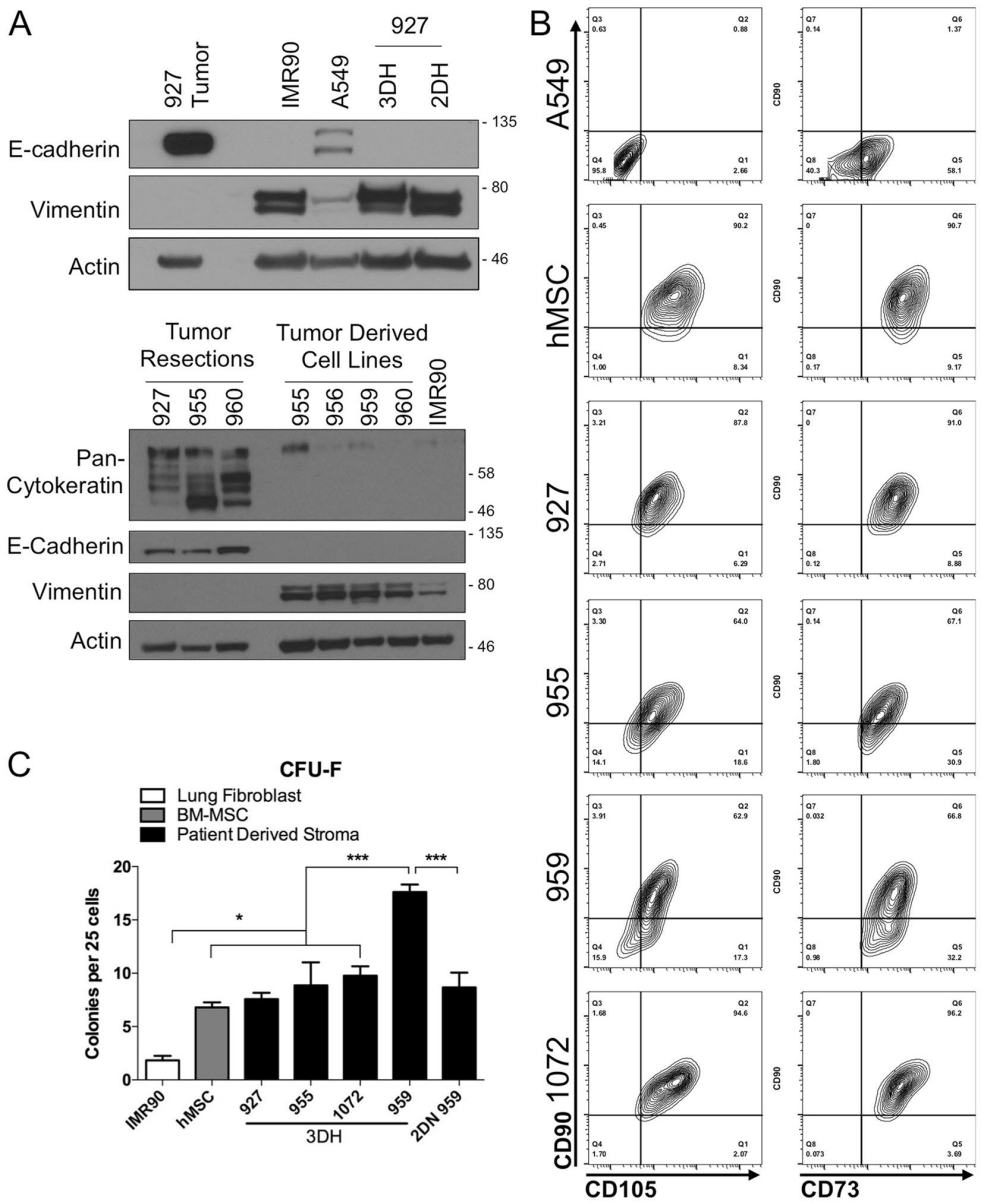
3DH Patient derived stromal cell lines are mesenchymal and possess mesenchymal stem cell-like characteristics. (**A**) Western blot analysis of tumor resection 927 and its tumor derived cell lines, fetal lung fibroblast cell line IMR90, and lung adenocarcinoma cell line A549 (upper). Western blot analysis of additional tumor resections and their derived cell lines probing for expression of pan-cytokeratin (lower). Cropped images in the figure are derived from full length blots presented in Supplementary Fig. S4. (**B**) Immunophenotyping of 3DH tumor derived cell lines with commonly identified antigens associated with MSC (Y-axis: CD90, X-axes: CD105 (left) and CD73 (right)). (**C**) Quantification of Colony Forming Unit-Fibroblast (CFU-F) per 25 cells plated in 3DH tumor derived cell lines, a matched 2DN derived cell line (2DN 959), bone-marrow derived mesenchymal stem cell line (hMSC), and IMR90 after 16–18 days of growth. Statistical analysis performed by ANOVA with Tukey’s test for multiple comparisons to the indicated groups, *p < 0.05, and ***p < 0.001; data presented are mean ± S.E.M.

While we observed a heterogenous population of cell morphologies in the first week of cell isolation (Fig. S2), the spindle-shaped cells quickly outpaced the other cell populations. We tested protein expression of epithelial and mesenchymal cell lineages and found that while the 927 tumor resection was highly positive for e-cadherin expression, cell lines derived from the resection were highly vimentin positive with undetectable e-cadherin expression (Fig. 3A). Similarly, cell lines derived from the other tumor resections did not express detectable levels of E-cadherin or Pan-cytokeratin but were also highly vimentin positive. Given the high proliferative capability and strict cell culture conditions utilized in the isolation, we suspected this cell type may be a mesenchymal stem cell. We measured surface expression of well characterized mesenchymal stem cell markers CD90 (Thy1), CD73 (NT5E), and CD105 (Endoglin) (Fig. 3B). The tumor derived cell lines expressed CD73 in the highest quantity, with the majority also staining positive for CD90 as well as CD105.

Mesenchymal stem cells are also defined by their ability to adhere to tissue culture plastic and form colonies from colony-forming unit-fibroblasts (CFU-F). Populations of CFU-F were significantly different between patient sample cell lines, IMR90 fetal fibroblasts, and hMSC (F (6,18) = 21.32, P < 0.0001) by one-way ANOVA. Upon multiple comparisons, all patient sample cell lines possessed significantly greater CFU-F populations compared to IMR90 fetal fibroblasts (P < 0.0314), however tumor sample 959 derived in 3DH was distinguished with significantly greater CFU-F compared to either hMSC or the other tumor samples (P < 0.0003). There was no significant difference in tumor samples 927, 955, or 1072 in colony forming ability compared to hMSC (Fig. 3C). Interestingly, the environment that tumor sample 959 was originally cultivated in had a significant effect (P < 0.0003) on maintenance of colony progenitors, where the 3DH environment maintained a greater quantity of progenitors compared to traditional 2DN conditions. Taken together, these findings suggest that culturing cells derived from NSCLC in a lung microenvironment mimetic environment rapidly selects for mesenchymal stromal cells with stem-like characteristics. Given the delay in growth in traditional conditions and that at least one of the samples showed a difference in progenitor characteristics depending on the environment in which it was derived, we asked whether cells cultivated from the same tumor in different environments were phenotypically similar.

### Environment dependent MSC multipotency markers

**Figure 4:**
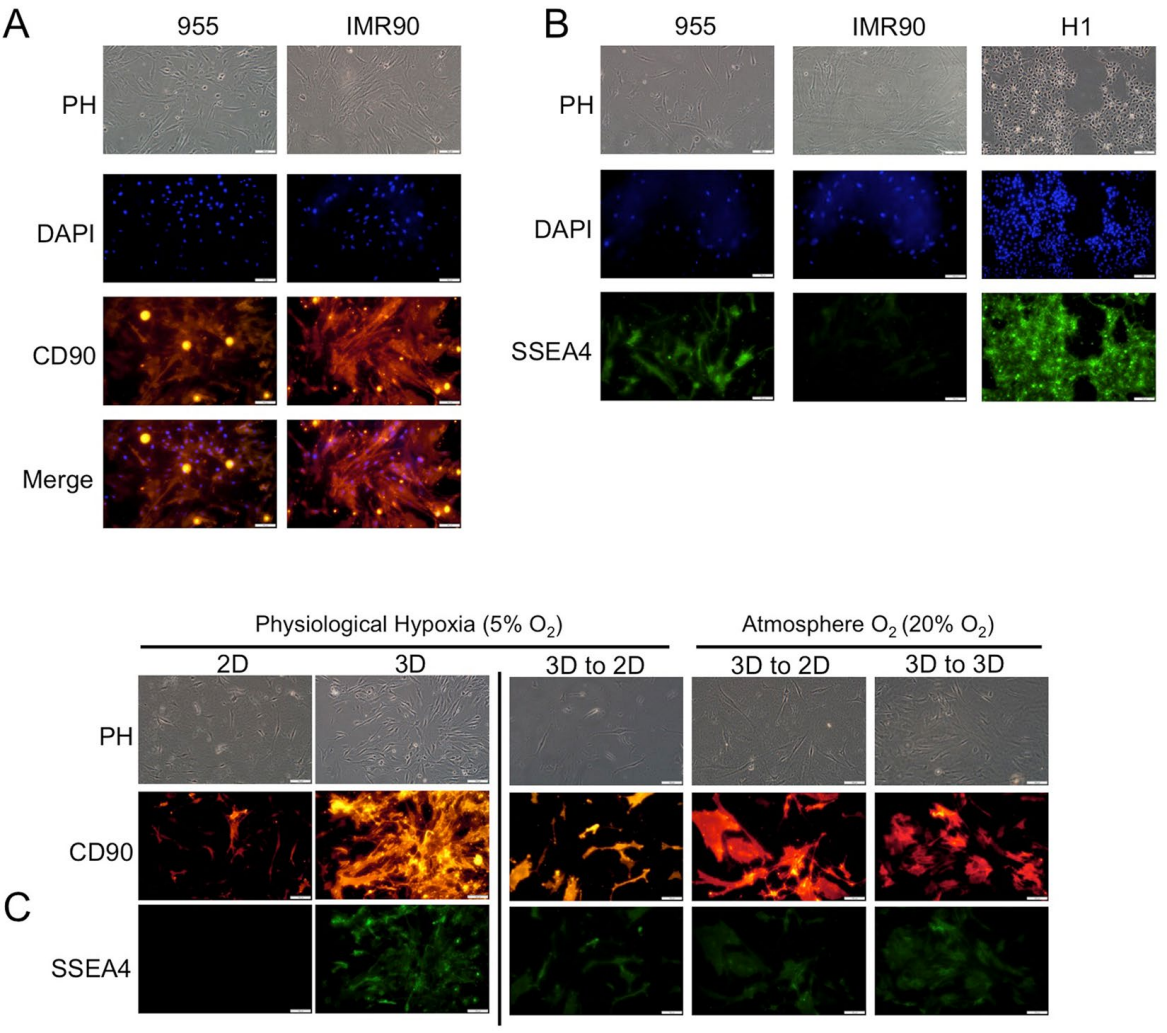
3D Physiological hypoxia cell culture selects for SSEA4 expressing tumor derived stromal cells. (**A**) Phase contrast and immunofluorescence staining of IMR90 lung fibroblasts and patient sample 955 patient tumor stromal cells for stromal marker CD90/Thy1 and nuclear stain DAPI. (**B**) Staining of stromal cells and human pluripotent embryonic stem cell line H1 for stage specific embryonic antigen-4 (SSEA4). (**C**) (left) SSEA4 expression of patient tumor stromal cell line 927 derived in either 2D or 3D hypoxia. (right) SSEA4 expression of patient tumor stromal cell line 927 3DH transferred to other environments. All scale bars are 100 *μ*m.

Mesenchymal stem cell markers and their differentiation capacity into various stromal cell types vary greatly depending on the sites and species from which they are derived, and the methodology used in their isolation^23^. However, human adult bone marrow mesenchymal stem cells, similarly to pluripotent human embryonic stem cells, were previously demonstrated to express a stage-specific embryonic antigen, SSEA-4^24^. To test whether primary cells isolated in the microenvironment mimetic environment maintained multipotent characteristics similar to MSC, we assessed SSEA-4 expression on sample 955 via immunofluorescent labeling. We confirmed that the majority of the cell population expressed CD90 (Fig. 4A), and also observed that most of the cells maintained SSEA-4 expression (Fig. 4B). We also tested sample 927, which only produced viable progenitor cell lines in hypoxic environmental conditions (2DH and 3DH). We found that only the sample that had proliferated in the microenvironment mimetic 3DH environment maintained strong expression of SSEA-4 (Fig. 4C). The 3DH cells were then passaged in the other environments for 72 hours, and each of the environments maintained SSEA-4 expression. This suggests that certain characteristics induced in the microenvironment culture may be maintained or the rate of expression reduction is slowed upon transfer to traditional cell culture methods.

**Figure 5:**
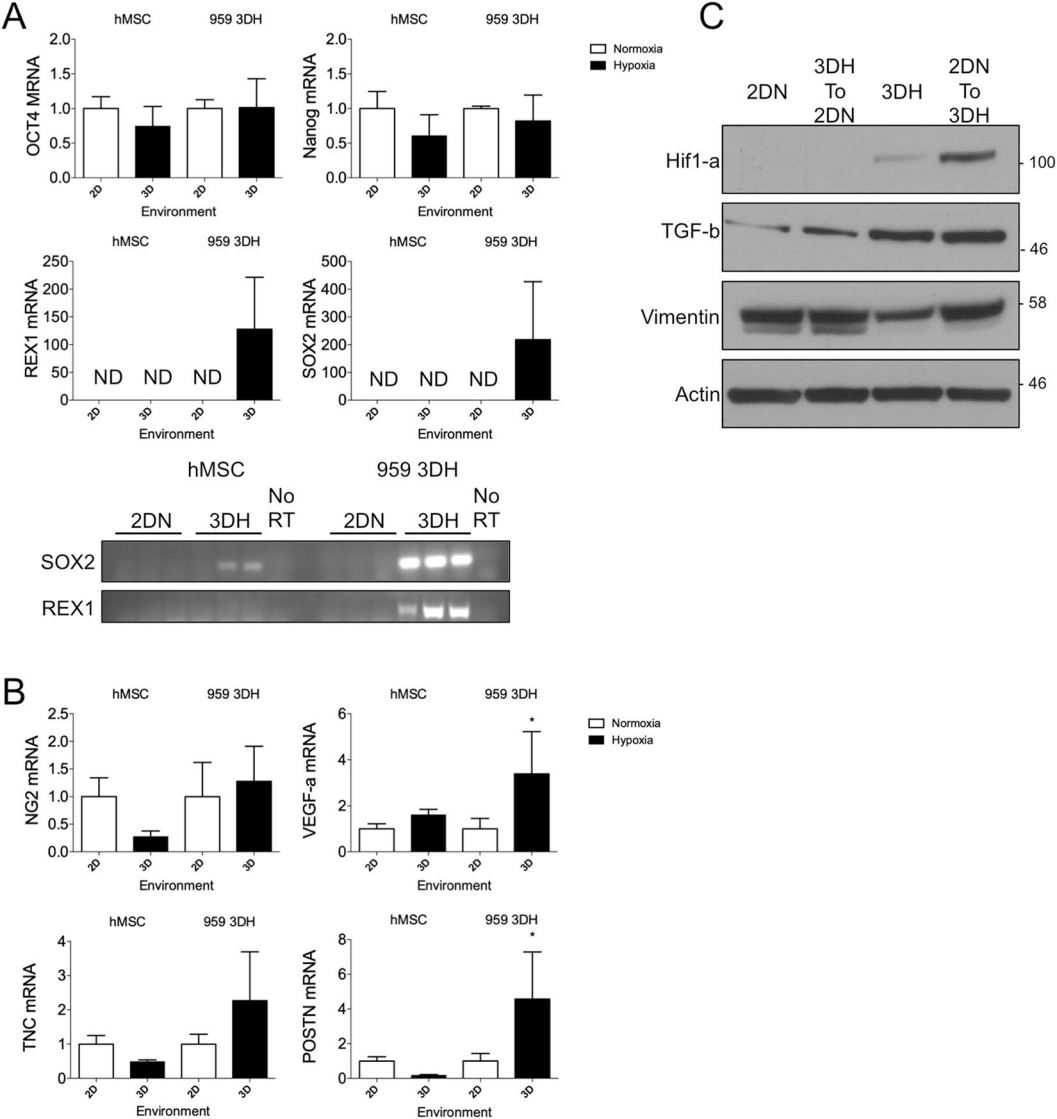
3D Physiological hypoxia cell culture maintains expression of pluripotency markers and tumor promoting characteristics of tumor derived stromal cells. hMSC and 3DH derived 959 were cultured in their native culture environments or transferred for 72 hours prior to collection of RNA and protein for RT-qPCR and western blot. (**A**) Relative expression levels of pluripotency markers assessed by RT-qPCR of hMSC and 3DH 959 in different environments. Non-detectable products were confirmed on agarose gel. Cropped images were derived from full length gels presented in Supplementary Fig. S5. (**B**) RT-qPCR of stromal derived tumorigenic factors of hMSC and 3DH 959 in different environments. (**C**) Western blot analysis of HIF1-a and TGF-b expressed in 2DN or 3DH derived 959 stroma and transferred to different environments for 72 hours. Cropped images in the figure are derived from full length blots presented in Supplementary Fig. S6. Statistical analysis performed by ANOVA with Sidak’s test for multiple comparisons to the indicated groups, *P < 0.05; data presented are mean ± S.E.M.

In order to verify that a physiological microenvironment indeed plays a role in the potency characteristics of derived cell lines, we assessed several markers of stem cell potency in hMSC and the 959 cell line that had been grown in the 3DH environment. Each cell line was split to both environments and allowed to grow for 72 hours before collection. Cells that were maintained in the 3DH environment expressed higher amounts of the markers SOX2 and REX1 (also known as ZFP42) compared to 2DN, but Nanog and Oct4 (also known as POU5F1) were not significantly affected (Fig. 5A). Interestingly, when the hMSC were transferred to the physiological condition, SOX2 and REX1 were undetectable. Therefore, a physiological microenvironment mediates potency characteristics of primary tumor derived stromal progenitor cells but may not be sufficient in enhancing potency of established cell lines.

### Tumor stromal cells derived in 3DH preserve tumor promoting characteristics

Given the possibility that we may have selected for an MSC subtype in our microenvironment mimetic culturing system, we aimed to understand whether our system generally activated MSC to become tumorigenic and whether tumor derived stromal progenitor cells also produced pro-tumorigenic factors. Thus, we assessed stromal genes related to worse prognosis and tumor progression in both cell types. Gene expression of VEGFA was significantly increased (P < 0.0368) in tumor stromal cells maintained in 3DH environment, but not greatly affected upon hMSC transfer to 3DH (Fig. 5B). Similarly, periostin (POSTN), a secreted extracellular matrix protein that plays a role in metastasis and is associated with a worse prognosis in a variety of cancers^25^, had significantly higher gene expression when tumor stromal cells were grown in 3DH (P < 0.0164) but was slightly decreased when hMSC were transferred to 3DH. Neuron-glial 2 (NG2, also known as CPSG4), an angiogenic pericyte protein associated with the pre-metastatic niche, and tenascin-C, an extracellular matrix protein frequently overexpressed in lung cancer, were not greatly affected by environment transfer in either hMSC or tumor stromal cells. Transforming growth factor beta 1 (TGF-*β*) was also increased in the 3DH environment, however when 3DH cells were transferred to 2DN they maintained slightly higher expression (Fig. 5C). Our findings suggest that only tumor derived cells cultured in microenvironment mimetic culture alter their phenotype to produce a variety of pro-tumorigenic factors, and we sought to test whether these factors might alter the phenotype of NSCLC cells themselves.

**Figure 6:**
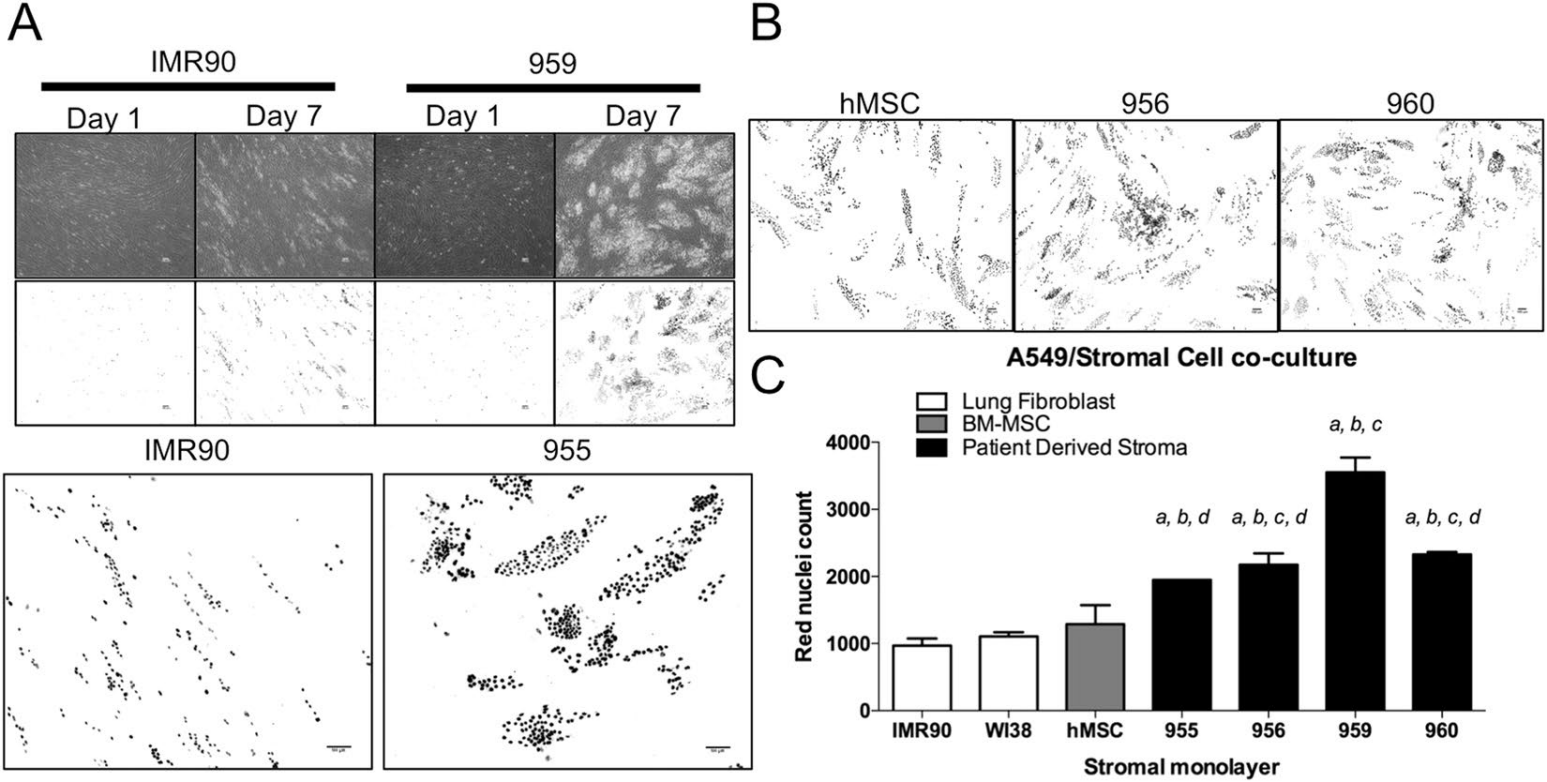
Cell-cell interactions between 3DH Patient derived stromal cell lines and lung adenocarcinoma promote cancer cell proliferation and colony formation. Stromal cells were grown to a confluent monolayer for four days, and then equal amounts of red nuclear labeled A549 cells were plated atop monolayers. (**A**) (Upper) Phase contrast and fluorescent images of A549 grown on stromal monolayers of IMR90 and patient stroma 959. Images obtained 1 and 7 days after cancer cell seeding. (Lower) Comparison of A549 colony formation on IMR90 or 955 monolayers on day 7. (**B**) Comparison of A549 colony formation on hMSC, 956, and 960 monolayers. (**C**) Quantification of red nuclei on day 7 (mean nuclear counts were obtained from multiple images of technical duplicate plates from 2 different experiments.) Statistical analysis performed by ANOVA with Tukey’s test for multiple comparisons to the indicated groups (a: vs. IMR90, P < 0.01; b: vs WI38, P < 0.05; c: vs. hMSC, P < 0.05, d: vs. 959, P < 0.01); data presented are mean ± S.E.M. All scale bars are 100 *μ*m.

To determine if direct interaction with patient derived stroma would alter cancer cell growth, we co-cultured fluorescent nuclear labeled A549 lung adenocarcinoma cells on different stromal monolayers. Following seven days of growth, A549 co-cultured with patient stroma rapidly expanded (Fig. 6A) and formed large, densely nucleated colonies compared to smaller, more linearly arranged growth on fetal lung fibroblast monolayers. This colony forming ability was also maintained on the MSC monolayer (Fig. 6B). Fluorescently labeled nuclei were quantitated and one-way ANOVA revealed that different stromal monolayers significantly affected the growth of A549 cells (F (6, 9) = 36.51, P < 0.0001). We found that there was no significant difference of cell growth on either IMR90, WI38, or hMSC monolayers, however all patient samples significantly increased cancer cell growth compared to lung fibroblast monolayers IMR90 and WI38 (955: P < 0.0088, P < 0.0495; 956: P < 0.0021, P < 0.0126; 959: P < 0.0001, P < 0.0001; 960: P < 0.0009, P < 0.0052). Three of the patient derived stromal monolayers significantly increased growth compared to hMSC (956: P < 0.0380; 959: P < 0.0001, 960: P < 0.149). Of the patient derived cell lines, 959 significantly increased cancer growth compared to the others (955: P < 0.008; 956: P < 0.0023; 960: P < 0.0053).

**Figure 7:**
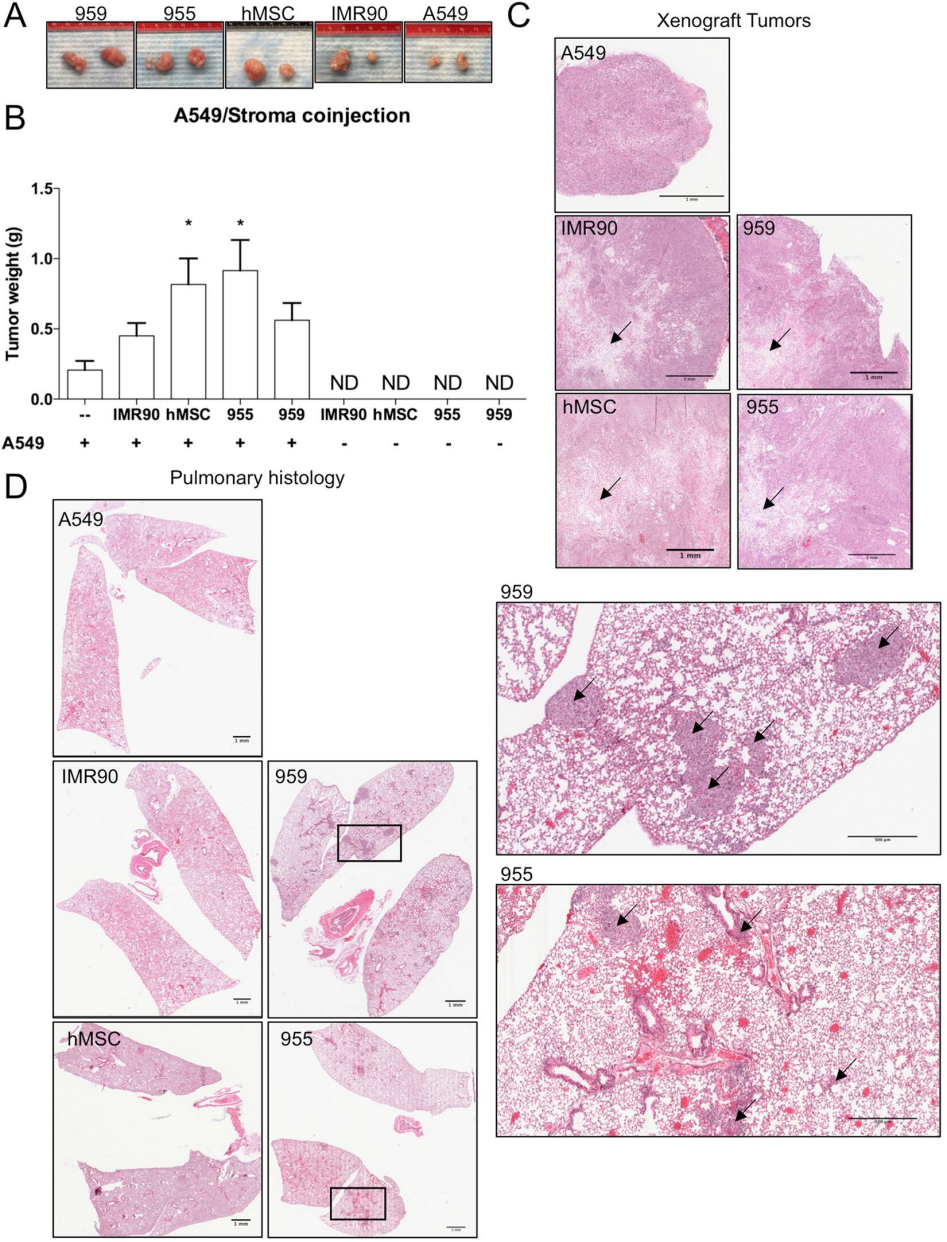
955 and 959 stromal cell lines were subcutaneously injected into alone or mixed with A549 cancer cells (5:1) and subcutaneously injected into flanks of NRG mice. IMR90 and hMSC co-injections served as controls. (**A**) Representative images of tumors excised after 30 days of growth. (**B**) Quantification of excised tumor weights (g) (statistical analysis performed with ANOVA and Tukey’s test for multiple comparisons vs. A549 injection alone, *p < 0.05; data represented as mean ± S.E.M.) (**C**). Histology of subcutaneous xenograft tumors. Arrows indicate areas of stromal cells; scale bars are 1 mm. (**D**) Pulmonary histology of lungs from mice receiving xenograft tumors (left; scale bars are 1 mM). 955/A549 and 959/A549 co-injection tumor bearing mice with pulmonary metastasis (inset; scale bars are 500 *μ*m). Arrows indicate metastatic lesions.

Patient stromal cell lines promote xenograft tumor growth and metastasis. Since we observed a significant increase of cancer cell growth upon direct interaction with patient tumor stromal progenitors, we sought to determine whether these cells isolated and grown in 3DH also displayed pro-tumorigenic ability *in-vivo*. To that end, we subcutaneously injected combinations of A549 lung adenocarcinoma cells and stromal cell lines, separately and co-administered (1:5), into flanks of immunocompromised NRG mice. After 30 days of growth, tumors successfully developed in all mice bearing A549 (Fig. 7A), whereas no tumors developed in any mice injected with only stromal cells (Fig. 7B). One-way ANOVA comparison of mean tumor weights revealed statistically significant differences in tumor growth with stromal co-administered tumors (F (4, 35) = 3.710, P = 0.0128). While generally the addition of stroma resulted in increased tumor growth, statistically significant increases in tumor weight compared to solely A549 injected tumors revealed these changes may primarily be driven by hMSC (P < 0.0450), and 955 (P < 0.0124) co-injections. Histological sections of the subcutaneous tumors revealed that stromal populations were present and localized toward the core of tumors in co-injections (Fig. 7C), but a prominent stroma was not evident in the solely A549 tumors. Pulmonary histological analysis revealed small metastatic nodules in IMR90 co-injected mice, but prominent metastatic lesions in hMSC, 955, and 959 co-injected cohorts (Fig. 7D). Pulmonary metastasis was not detected in solely A549 injected mice. In summary, mesenchymal stromal stem-like cells derived from early, non-metastatic tumor resections in a microenvironment mimetic environment maintain tumor promoting characteristics *in-vitro* and promote a full metastatic program in a normally non-metastatic adenocarcinoma cell line xenograft.

## Discussion

Previously, we have shown that lung fibroblast derived extracellular matrices provide various benefits to lung adenocarcinoma cell lines *in-vitro* such as providing resistance to apoptosis, serum withdrawal and chemically induced hypoxia. Since the environment is a critical determinant of cell fate in NSCLC, we expanded our *in vitro* system to include a physiological oxygen tension that would resemble the microenvironment of the lung. Initially, we sought to test each individual element of our proposed microenvironment mimetic culturing system (Fig. 1A) and found that the individual components of hypoxia (2DH) or cell derived ECM (3DN) enhanced mesenchymal cell growth after 72 hours (Fig. 1B). Interestingly, the combination of hypoxia and ECM appeared to have the greatest effect on cell growth. Others have demonstrated the mitogenic benefits of low levels of hypoxia (1–5% O2) to multiple mesenchymal cell types. Grayson *et al*. showed that hMSC grown in 2% O2 improved their cell proliferation after extended periods but did not enhance their proliferation rate^22^. Instead, they concluded that hypoxia prolonged the proliferative state and the cells were less affected by inhibitory signaling. Our analysis of cell doubling time agreed with their finding as we did not observe significant differences in doubling time for hMSC in hypoxia, and only IMR90 fetal lung fibroblasts had a significantly hastened proliferation rate in hypoxia (Fig. 1C). However, all cell lines tested exhibited a significant reduction in doubling time when cultured in the combination of physiological hypoxia and ECM (3DH), suggesting that there may be an increase in proliferation kinetics and cell cycle progression. This is supported by the relative decrease in cyclin-dependent kinase inhibitors p21-Cip1 and p27-Kip1 in 3DH grown cells compared to 2DH (Fig. 1D). This may yet highlight another benefit of microenvironment mimetic culturing, as these factors, as well as effects mediated by P53 and Akt, play important roles in the induction of cell senescence, a major challenge in the culture and expansion of human mesenchymal progenitor cells and fibroblasts^26–28^. Indeed, we found that most cell lines tested continued to proliferate rapidly for more than 15 passages (Fig. S2). It is possible that the combined decrease in inhibition of cell cycle progression with the increased 3-dimensional surface area allotted by the ECM act synergistically to allow proliferation at high cell densities, resulting in a sustained proliferative response.

Given this sustained proliferative response, and the reported capability of ECM derived from desmoplastic and tumor conditioned fibroblasts to induce desmoplastic differentiation in stromal cells grown on those matrices^8^, we also sought to determine if our system was sufficient to promote desmoplastic differentiation of stroma. In accordance with their finding, our fibroblast derived matrix system did not induce expression of a desmoplastic response in normoxia or hypoxia (Fig. 1D). In addition, we found that cells grown in 3DH had less HIF1-a stabilization compared to their 2DH counterpart, indicating a role for the ECM in the regulation of the oxygen sensing response and homeostasis. Taken together, these data provided evidence that stromal cells would receive a proliferative benefit in a microenvironment mimetic system and that the system may not artificially accelerate the tumorigenic potential of that stroma.

Next, we obtained small tumor resections from five treatment naïve patients diagnosed with NSCLC (Table 1 and Fig. S2). A small portion of these samples was dissected, enzymatically digested, and equal quantities of cells in single cell suspensions were aliquoted into separate environmental conditions. The first of these samples, 927, was tested in each condition (2DN, 2DH, 3DN, 3DH). After 7 days of growth, we observed cell attachment in every condition (Fig. 2A), and while there was increased cell number in 2DH and 3DN, the 3DH condition had a substantial increase in cells and numerous large colonies covering the plate. Of the environments tested, only the hypoxia environments were capable of cultivating cell lines. Due to the marked increase in long term cell viability and the limited quantities of our patient samples, we chose to consolidate our testing to traditional cell culture methods (2DN) or microenvironment mimetic methods (3DH). Further sample isolations confirmed our previous results, with every sample tested yielding very rapid expansion of cells into cell lines in 3DH compared to 2DN (Fig. 2B and D). For one biological specimen, we were also able to acquire adjacent normal tissue, however we did not observe the same growth benefit in 3DH (Fig. 2C). It is possible that the number of progenitor cells present in this tissue was less than that which resides within the tumor microenvironment and was unable to sustain a population.

Alternatively, stromal progenitor cells within the tumor microenvironment may also undergo metabolic reprogramming to allow them to adapt more easily to a hypoxic cell culture system^12^. Considering that we were only able to test one specimen, it is difficult to draw conclusions and further studies are required to determine the viability of 3DH culture in isolating primary human tissue resident stromal progenitors. Importantly, while traditional methods produce cell lines after a delay, 3DH isolation increases cell viability of tumor derived stroma, and cells isolated in this environment can be transferred to traditional cell culture (Fig. 2E), although at a slowed proliferation rate, allowing for testing via common pre-clinical research techniques.

It should be noted that initially every sample allowed for the attachment of a heterogenous population of cell morphologies (Fig. S2), and some of these cell types were present and maintained their *in-vivo* cell polarity characteristics upon passaging in 3DH (Movie S1). This suggests that our microenvironment mimetic platform can be further manipulated to preserve other populations of cancer stem or progenitor cells, perhaps with supplementation of specific growth factors. Nonetheless, the majority cell type that proliferated was of mesenchymal origin (Fig. 3A) and possessed surface expression of common markers for human mesenchymal stem cells (Fig. 3B). Recently, there has been some discussion in the field of the tumor associated stroma regarding the hypothesis that cancer associated fibroblasts (CAF) may actually be quiescent mesenchymal stem cells that become activated within the tumor^12^. This is supported by research showing that breast cancer derived CAF are remarkably similar to hMSC in their morphology, surface marker expression, cytokine production, and trilineage differentiation potential^29^. The isolation and cultivation of CAF and hMSC is also similar and dependent upon adherence to tissue culture plastic either from single cell suspensions or from cells that migrate from dissected tissue fragments. When comparing the CFU efficiency of the isolated cell lines, they were similar to hMSC (Fig. 3C), however the efficiency of at least one cell line appeared to be significantly higher when it was isolated in 3DH compared to traditional methods. It is possible that cells isolated with traditional methods possess MSC-like characteristics but gradually lose them and progenitor capability over time, whereas cells isolated and maintained in 3DH preserve those characteristics.

Multipotency is one of the defining characteristics of hMSC^30^. Markers associated with potency are an active area of research^31^, and these markers vary depending on the species, age, and tissue of origin of the isolated cells^32,33^. We found that tumor stromal progenitor cells cultured in 3DH expressed SSEA4 (Fig. 4B), similarly to hMSC derived from bone marrow. We did not observe expression of SSEA4 in 2DN derived samples, however SSEA4 was detectable when the 3DH derived cells were transplanted to other environmental conditions. We hypothesize that the 3DH condition selects for and rapidly amplifies the SSEA4 positive population, whereas this population grows at a delay in standard culture conditions before amplifying, similarly to unsorted bone marrow samples examined by Gang *et al*.^24^. 3DH derived cells also expressed pluripotency markers Oct4, Nanog, Rex1, and Sox2 (Fig. 5A), however only Rex1 and Sox2 appeared to be dependent upon the environmental condition of the cells. This corresponds with earlier studies of bone marrow MSC and embryonic stem cells grown in hypoxia^34,35^. Considering these data and that ECM produced by bone marrow MSC plays a pivotal role in their maintenance of stem-like characteristics^15^, the combination of a native ECM and hypoxia seems to isolate multipotent progenitor cells from the pulmonary tumor microenvironment and that these cells may be MSC.

It is well known that MSCs from the bone marrow have a remarkable capacity to home to tumors of a variety of different organs and affect their growth, as well as their response to treatment. While it is currently not possible to definitively identify whether the progenitor cells in the patient tumors we studied were tissue-resident or bone marrow derived, we asked the question of whether these tumor associated MSC had different tumor promoting effects compared to hMSC. We found that the 3DH environment significantly increased gene expression of angiogenesis promoting VEGF-A^36,37^ and metastasis promoting periostin^25,38^ in a patient derived cell line, but not in hMSC (Fig. 5B), and also increased TGF-b expression even after the cell line was transferred back to the 2DN condition (Fig. 5C). Patient stroma also significantly increased A549 lung adenocarcinoma cell growth in a direct co-culture model *in-vitro* compared to hMSC and fetal lung fibroblasts (Fig. 6C). Interestingly, A549 clustered into dense nests of cells when cultured on hMSC or patient stromal monolayers, but not on the fibroblast monolayers (Fig. 6A,B). These cell nests, as well as nests observed in early culture of NSCLC derived cell lines (Fig. S2), resembled sphere forming cancer stem cells(CSC) induced in a CSC/CAF co-culture system^39^. It is possible that the microenvironment mimetic culture method also plays a role in establishment or maintenance of CSC populations, especially in the context of additional paracrine signaling with tumor stroma. To understand these interactions *in vivo*, xenograft studies demonstrated that subcutaneous co-injection of A549 with stroma significantly increased tumor size compared to A549 alone (Fig. 7A,B), and that this stroma remained present and localized toward the core of the tumor (Fig. 7C). Further, both cohorts of mice bearing patient stromal tumors developed metastasis to the lung, similarly to hMSC injected mice while not in A549 alone (Fig. 7D). This suggests that patient stroma is sufficient to promote a normally non-metastatic cancer cell line to undergo the full metastatic process. Whether this effect is due to the increased expression of stromal TGF-b, VEGF-A, periostin, or another stromal derived factor remains to be elucidated.

In toto, this study demonstrates the benefits of an *in-vitro* culturing system that mimics two important elements of the tumor microenvironment, physiological oxygen tension and native extracellular matrix structure, in the isolation of stromal progenitor cells from lung tumor biopsies. These progenitor cells express some commonly used markers of stem cells within our cell culture system, and function similarly to hMSC in supporting an *in vitro* tumor-like environment. We realize that many cells express these markers and more studies are needed to fully characterize their specific identity, and given our data, especially the potential role they may play in supporting a purported cancer stem cell phenotype. This work further enables the clinically relevant study of patient specific tumor features that are sufficient to promote tumor growth and metastasis. Indeed, our findings highlight the importance of investigation using *in vitro* models that reflect the *in vivo* condition as several tumor promoting characteristics were revealed only in physiological hypoxia and on extracellular matrix. Targeting factors that are producible in a physiological model may yet lead to greater drug targeting efficacy for future anti-tumor and anti-tumor associated stroma therapy. Additionally, this methodology may be refined to provide further insight into progenitor cells of other tissues and assist in rapidly expanding stem populations and preserving multipotency.

## Methods

### Human tissue samples

Primary human lung cancer resections (six) were acquired and de-identified from patients undergoing resection of pulmonary tumors without preoperative chemotherapy or radiotherapy at the James Graham Brown Cancer Center at the University of Louisville. Patients with lung adenocarcinoma and squamous cell carcinoma were staged according to the tumor-node-metastasis system established by the American Joint Committee on Cancer. This study was approved by the IRB committee of the University of Louisville (#130188) and informed consent was obtained from all patients. All experiments were performed in accordance with relevant guidelines and regulations.

### Hypoxic cell culture

Hypoxic cell culture conditions (5% O_2_, 5% CO_2_, 90% N_2_, 37 °C) were achieved with a modified water jacketed cell culture incubator equipped with a ProOx P110 oxygen controller (BioSpherix, Parish, NY). The sensor was calibrated and programmed to maintain a setpoint of 5% O_2_ via infusion of Ultra High Purity Nitrogen gas.

### Patient sample processing

Tumor resections were transported on ice, transferred to a sterile 100 mm cell culture plate in ice-cold PBS, imaged and placed in a sterile laminar flow hood. The tissue was diced to approximately 1 mm^3^ pieces. The diced tissue was transferred to a vial containing collagenase/dispase (Sigma, St. Louis, MO) dissolved (1 mg/mL) in serum free Dulbecco’s modified Eagle’s medium-high glucose (DMEM) for enzymatic digestion. The vial was warmed to 37 °C and incubated with rocking for at least 1 h 15 m. The tissue suspension was agitated with vigorous pipetting to confirm tissue dissociation and either returned to 37 °C if incomplete or inactivated. Cell suspensions passed through a 70 *μ*m cell strainer. A syringe plunger was used to force remaining tissue through the strainer and the strainer washed with ice cold PBS. The tissue suspension was cooled to 4 °C and then placed in a centrifuge for 3 minutes at 400 cfg and 4 °C. The supernatant was aspirated and the cell pellet suspended in growth medium (DMEM 2% FBS, Pen/Strep, Cipro). Cell and viability counts were obtained with a TC20 automated cell counter (Bio-rad), and cells were placed onto a plate coated with human cell derived extracellular matrix and maintained in a climate-controlled incubator at 37 °C, 5% CO_2_, and 5% O_2_ (Fig. 1A). Cells were monitored for attachment and healthy morphology at 24 hours post culture and the media was changed 4 or 7 days post culture depending on cell attachment.

### Cell culture

Human normal fetal lung fibroblast cell lines WI38 and IMR90 were purchased from American Type Culture Collection (ATCC) and maintained in Minimum Essential Medium Alpha modification (MEM-*α*) supplemented with L-Gln, ribo- and deoxyribonucleosides (Fisher), 10% fetal bovine serum (Invitrogen) and 1% antibiotic/antimycotic (Sigma). Human bone marrow mesenchymal stem cells (hMSC) were purchased from Lonza and cultured as the fetal lung fibroblast cell lines. Human embryonic stem cell line H1(WiCell, Madison, WI) was cultured on human embryonic stem cell-qualified Matrigel-coated plates (BD Biosciences, San Jose, CA) in mTeSR1 media (STEMCELL Technologies, Vancouver, Canada), with media changed daily. All assays involving comparisons between tumor derived stromal cell lines (927, 955, 956, 959, 960, and 1072) and mesenchymal cell lines (WI38, IMR90, and hMSC) were performed with early passage cells (p < 8) in the same culturing medium, MEM-*α*, described previously. A549 NucLight Red human adenocarcinoma cells (Essen Bioscience) were cultured in RPMI medium supplemented with 10% FBS, 1% antibiotic/antimycotic, and 1 *μ*g/mL puromycin. All cell lines were maintained at 37 °C and 5% CO_2_, unless otherwise stated.

### Extracellular Matrix Preparation

Extracellular matrix preparation was performed as previously described^16^. Briefly, IMR90 fetal lung fibroblasts were plated at confluence in matrix growth medium (MEM-*α*, 10% FBS, 1% antibiotic). On the second, fourth, and sixth days, medium was supplemented with 50 *μ*g/mL L-Ascorbic acid (Sigma) to promote matrix deposition. On the eighth day, wells were washed with phosphate buffered saline (PBS) and cells were lysed with a detergent (20 mM NH4OH, 0.5% Triton-X-100) for 1–3 minute incubation at 37 °C until complete cell lysis was observed. Resulting cell derived matrices were gently washed three times with PBS, and then incubated with Dnase1 (10 U/mL) for 30 minutes to remove residual DNA. The matrices were then washed twice in PBS, and either used immediately or stored at 4 °C in PBS supplemented with 1% antibiotic for up to 4 months.

### Microscopy and immunocytochemistry

Phase microscopy images were obtained using a Zeiss AX10 inverted microscope. A549, H1, and patient derived cells were fixed with 2% PFA/PBS (10 minutes, 24 °C; Electron Microscopy Sciences, Hatfield, PA), permeabilized when needed with 0.05% Triton X-100/PBS (5 minutes, 24 °C; Sigma Aldrich, St. Louis, MO), and blocked with 5% normal goat serum/PBS (Sigma-Aldrich, St. Louis, MO). Cells were probed with mouse monoclonal antibodies recognizing SSEA-4 (1:200, ThermoFisher, Walton, MA) and CD90 (PE) (1:200, BD Biosciences, San Diego, CA) overnight at 4 °C. Cells were washed with PBS and incubated with goat anti-mouse 488 (1:1000, ThermoFisher, Walton, MA). DAPI nuclear stain (1:2000; ThermoFisher, Walton, MA) was added (5 minutes, 24 °C), washed, mounted, and imaged using Olympus IX81 fluorescence microscope (Center Valley, PA). Immunofluorescent images of co-culture experiments with red nuclear A549 were acquired with a Nikon Eclipse Ti microscope with a 4X objective using NIS-Elements AR acquisition software (Nikon Instruments, Inc., Melville, NY, USA). Nuclear counts from equal image thresholds were quantified automatically using the NIS-Elements AR software.

### Cell Proliferation, CFU-F, and Co-culture

Doubling time experiments were performed by plating approximately 4 × 10^4^ cells in technical duplicates. Mean cell counts were collected at 24 and 96 hours while cells were in log phase growth counting at least 100 cells in a hemocytometer. Doubling time was calculated using the formula DT = T ln2/ln(Xe/Xb), where T is the incubation time, Xb is the cell number at 24 hours, and Xe is the cell number at 96 hours. CFU-F assay was performed by seeding 50 cells in a 35-mm well. Media was refreshed on days 7 and 10. Colonies were fixed in methanol and acetic acid (7:1) on days 14–18 and were stained with crystal violet. Greater than 32 cells were considered colonies. For co-culture studies, stromal cells that had grown in 3DH (Fig. 1A.) were seeded at confluence density on 35 mm glass bottom tissue culture dishes (MatTek) to produce monolayers. After 4 days growth, 1.5 × 10^4^ Red A549 cells were seeded onto stromal monolayers and maintained in RPMI (10% FBS, 1% antibiotic/antimycotic) and monitored daily.

### Western Blot

Cells and tissue samples were harvested in CHAPS lysis buffer (1% CHAPS detergent, 150 mM NaCl, 50 mM Tris pH7, 5 mM EDTA) supplemented with cOmplete protease inhibitor and PhosSTOP phosphatase inhibitor cocktail tablets (Sigma-Aldrich, St. Louis, MO). Tissue samples were homogenized in a mini-bead beater. Protein was quantified by using Pierce’s BCA Protein Assay Reagent Kit (ThermoFisher, Walton, MA) as per the manufacturer’s instructions. Total protein (20 *μ*g) was heated at 95 °C for 5 minutes and separated by 4–12% SDS-polyacrylamide gel. Blots were transferred to PVDF membranes for 120 minutes at a constant voltage of 100 V in 4 °C. Membranes were blocked in 5% milk (w/v) in Tris-buffered saline Tween-20 (TBS-T) at 24 °C for 1 hour. The membranes were incubated with primary antibody in 5% milk TBS-T overnight at 4 °C. A list of antibodies used for analysis is presented in the Supplementary Material.

### Flow Cytometry

Cultures of A549, hMSC, and patient sample cell lines were collected with trypsin and washed with PBS. Cells were blocked for 15 min at 4 °C with Fc Block (#553142, BD Biosciences, Miami, FL, USA). The samples were then stained with a mix of fluorophore-conjugated anti-human monoclonal antibodies: FITC CD90 (Thy1), PE CD105, and PerCP/Cy5.5 CD73 (Ecto-5′-nucleotidase) from BioLegend (San Diego, CA) for 1 h at 4 °C. Stained cells were then examines on a Becton Dickinson FACScan with FlowJo analysis software.

### RT-qPCR

Total RNA from cell culture samples was isolated with E.Z.N.A Total RNA Kit (Omega), according to the manufacturer’s instructions. Total RNA (350 ng) was then DNase digested with TURBO DNA-free kit (Invitrogen) per the manufacturer’s instructions. DNase digested RNA was then reverse transcribed using High Capacity cDNA Reverse Transcription Kit (ThermoFisher, Walton, MA). Gene-specific cDNA was quantified with real-time RT-PCR using self-designed SYBR assays. Primers for qPCR were manually designed with Primer Express 3.0 software (Applied Biosystems, Foster City, CA) per the manufacturer’s instructions for SYBR green dye assays. RT-qPCR was performed with iTaq Universal Sybr Green Supermix (Bio-Rad). Expression levels were analyzed using ΔΔCT method and were normalized to *β*2-microglobulin (B2M) mRNA levels. PCR reactions were analyzed on a BioRad CFX96. Primer sequences and efficiencies are presented in Supplementary Material.

### Tumor Xenograft

NRG (NOD/RAG1/2^−/−^ IL2R *γ*^−/−^, #007799) mice were purchased from the Jackson Laboratory and animal procedures were approved by the institutional animal care and use committee under IACUC #18214 and all experiments were performed in accordance with relevant guidelines and regulations. 2.5 × 10^6^ IMR90, hMSC, 955, and 959 stromal cells were subcutaneously injected alone (N = 3) or co-injected with 5 × 10^5^ Red A549 (N = 8) bilaterally into the flanks of NRG mice. Mice were euthanized after 30 days and tumors and lungs were resected from mice. Tissues were fixed in 10% buffered formalin phosphate and paraffin embedded. Tissue sections were stained with H&E.

### Statistics

Student’s t-tests were used for comparisons between two groups. A one-way ANOVA with Tukey’s test for multiple comparison were used to determine statistical significance for multiple groups, unless otherwise stated. Statistical significance was considered for P-value < 0.05.

## Supplementary information

supplementary movie 1

supplementary info

## Data Availability

All data generated or analysed during this study are included in this published article (and its Supplementary Information files).
