# Supplementary material for: Primary lung cancer samples cultured under microenvironment-mimetic conditions enrich for mesenchymal stem-like cells that promote metastasis
Source: Sci Rep. 2019 Mar 12;9:4177. doi: 10.1038/s41598-019-40519-4 (PMC6414636; doi:10.1038/s41598-019-40519-4)
Supplement: Supplementary file 2 — supplementary info [file 41598_2019_40519_MOESM2_ESM.pdf]

Primary lung cancer samples cultured under microenvironment-mimetic conditions enrich for mesenchymal stem-like cells that promote metastasis

Douglas Saforo[1], Linda Omer[2,3], Andrei Smolenkov[4], Aditya Barve[1], Lavona Casson[4], Nolan Boyd[4,5], Geoffrey Clark[1,4], Leah Siskind[1,4*], and Levi Beverly[1,4,6*]

[1]University of Louisville, Department of Pharmacology & Toxicology, Louisville, KY, 40202, USA
[2]University of Louisville, Department of Biochemistry & Molecular Genetics, Louisville, KY, 40202, USA
[3]University of Louisville, Cardiovascular Innovation Institute, Louisville, KY, 40202, USA
[4]University of Louisville, James Graham Brown Cancer Center, Louisville, KY, 40202, USA
[5]University of Louisville, Department of Physiology, Louisville, KY, 40202, USA
[6]University of Louisville, Department of Medicine, Louisville, KY, 40202, USA
[*]leah.siskind@louisville.edu and levi.beverly@louisville.edu

Table S1. Antibodies and RT-qPCR primers

Antibody	Host	Catalog	Distributor
HIF-1α (D2U3T)	Rabbit	#14179	Cell Signaling Technologies
p21 Waf1/Cip1 (12D1)	Rabbit	#2947	Cell Signaling Technologies
α-Smooth Muscle Actin	Rabbit	#14968	Cell Signaling Technologies
Vimentin (D21H3)	Rabbit	#5741	Cell Signaling Technologies
E-Cadherin (24E10)	Rabbit	#3195	Cell Signaling Technologies
TGF-β	Rabbit	#3711	Cell Signaling Technologies
Basic Cytokeratin (AE3)	Mouse	#57004	Santa Cruz
p27 Kip1	Mouse	#610242	BD Biosciences
β-Actin	Mouse	#A2228	Sigma
Rabbit IgG HRP	Goat	#7074	Cell Signaling Technologies
Mouse IgG HRP	Horse	#7076	Cell Signaling Technologies

Gene	Forward	Reverse	Efficiency (%)
ZFP42 (REX-1)	TTTGAGTCCTTGGAATACCTAAAGAAA	AATTCTCTCCAACTATCTTTTGTGGAA	93.69
TNC (Tenascin C)	GAGAACAAGAAGAGCATTCCTGTCA	GGACTTGAATTTCAGGCCTTCA	100.29
POSTN (Periostin)	TGGTTATATGAGAATGGAAGGAATGA	GTCAGAATAGCGCTGCGTTGT	97.47
NANOG	GAACCTCAGCTACAAACAGGTGAA	TTTCTGCCACCTCTTAGATTTCATT	95.11
POU5F1 (Oct4)	CCAAGCTCCTGAAGCAGAAGA	TTGGCTGAATACCTTCCCAAA	96.03
SOX2	GCACAACTCGGAGATCAGCA	TCTCCGTCTCCGACAAAAGTTT	97.16
CPSG4 (NG2)	CACTGTCCTGCCTGTCAATGA	CCGTCCGTGCTCCTCAGA	91.39
VEGFA	CTTGCTGCTCTACCTCCACCAT	GACATCCATGAACTTCACCACTTC	107.09
B2M	TGACTTTGTCACAGCCCAAGATA	AATGCGGCATCTTCAAACCT	93.22

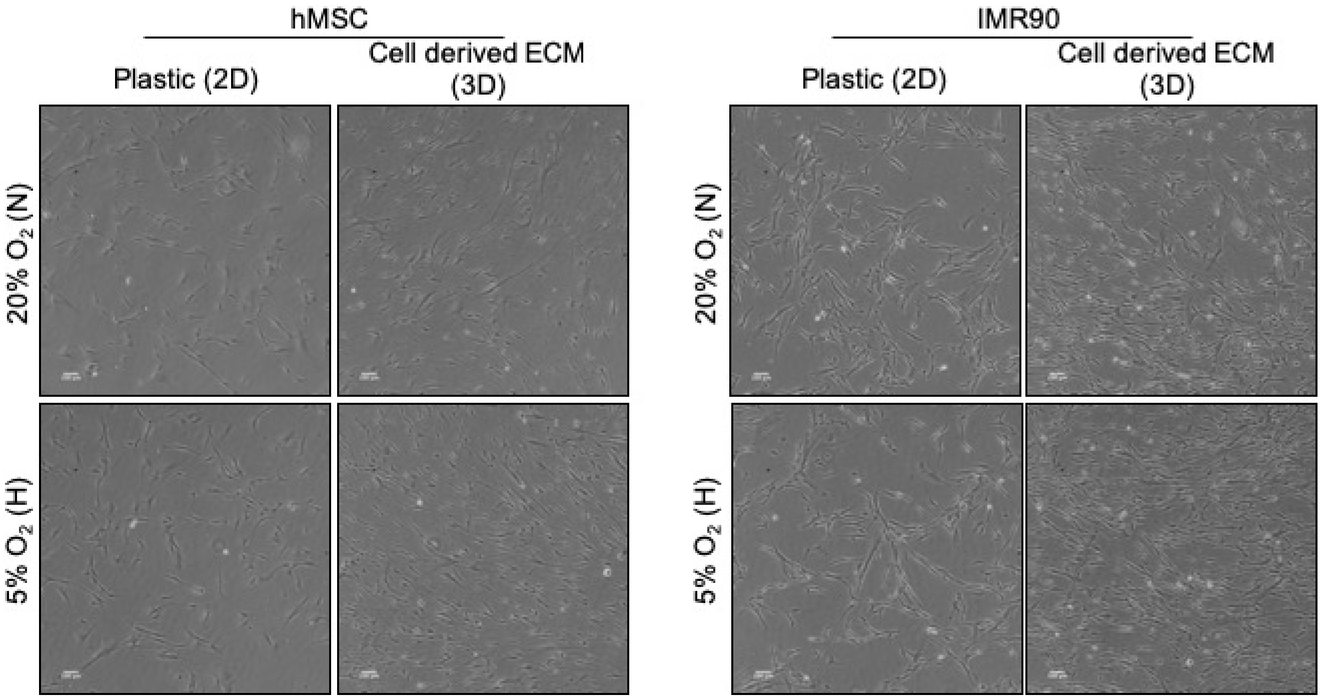

Figure S1. Phase contrast images of equal numbers hMSC and IMR90 fetal lung fibroblast cells grown for 72 hours in four environmental conditions (2D Normoxia, 2D Hypoxia, 3D Normoxia, and 3D Hypoxia). Scale bars are 100μM.

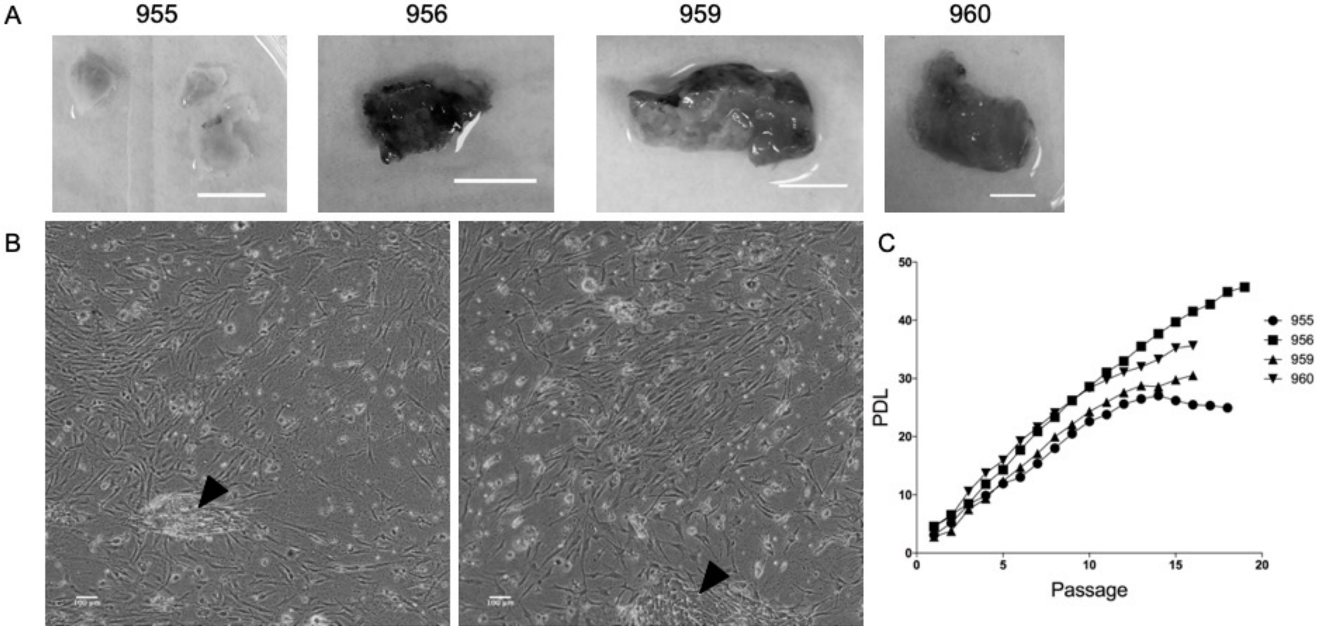

Figure S2. A. Images of patient tumor resections prior to dissection and dissociation. Scale bars are 1 cm. B. Phase contrast images of dissociated tumor cell growth from sample 956 7 days post isolation showing a heterogenous cell population. Arrowheads indicate clumps of non-fibroblastic colonies. Scale bars are 100μM. C. Population doubling level of cell lines derived from patient tumors and cultivated in 3DH.

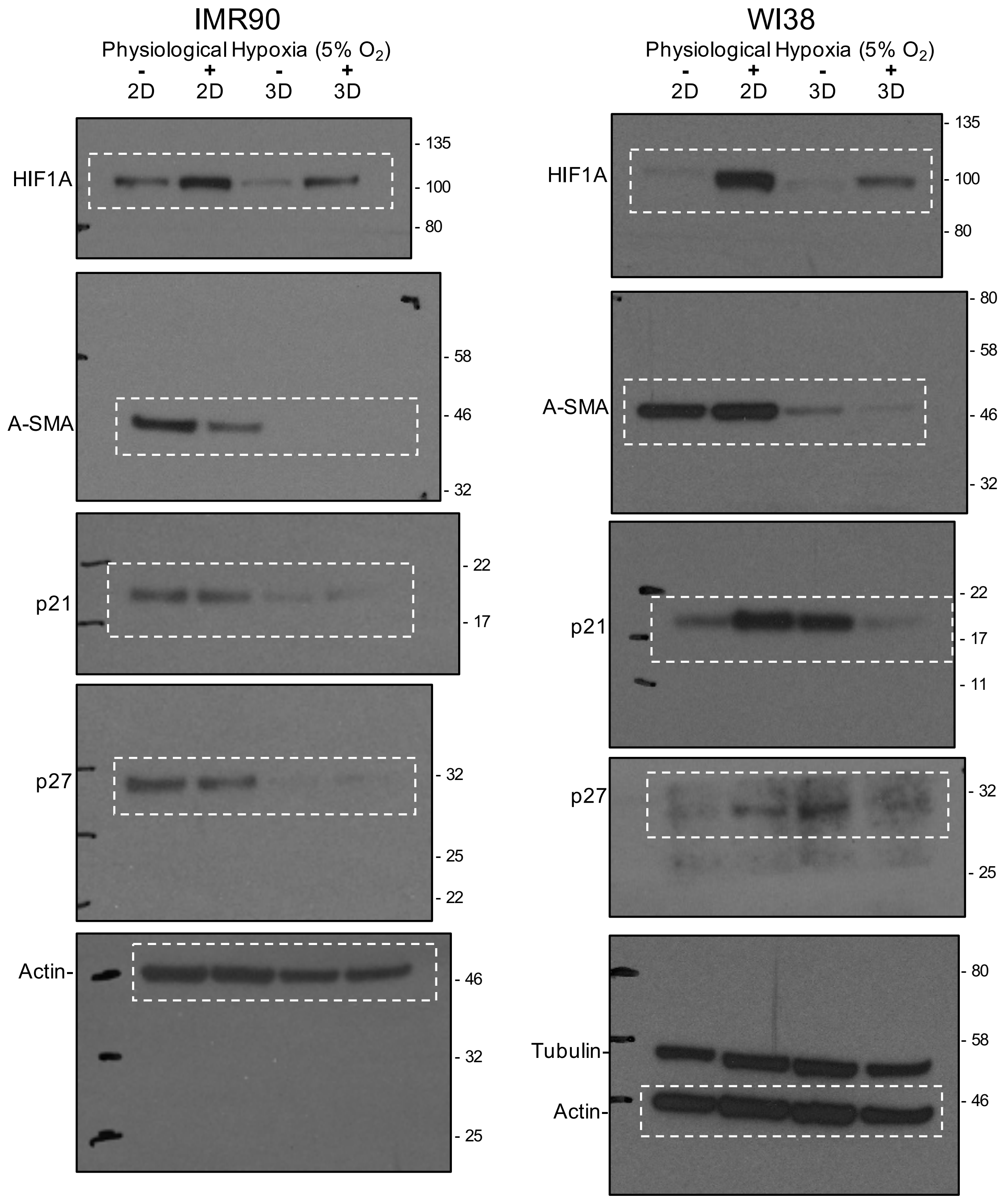

Figure S3. Full length blots of Figure 1D. White dashed lines indicate cropped areas. Brightness was adjusted in processing IMR90 blots of p21, p27, and actin.

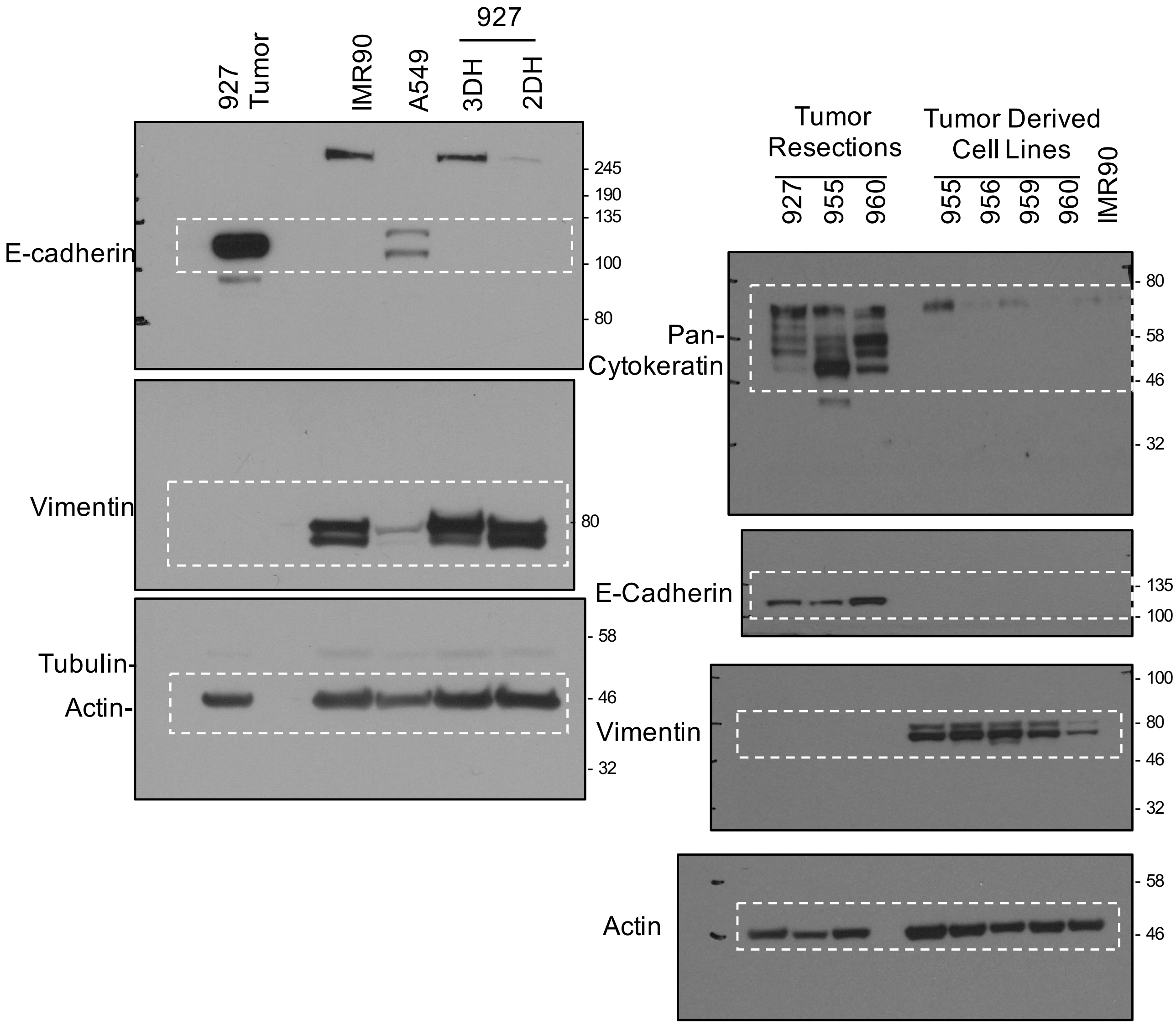

Figure S4. Full length blots of Figure 3A. White dashed lines indicate cropped areas.

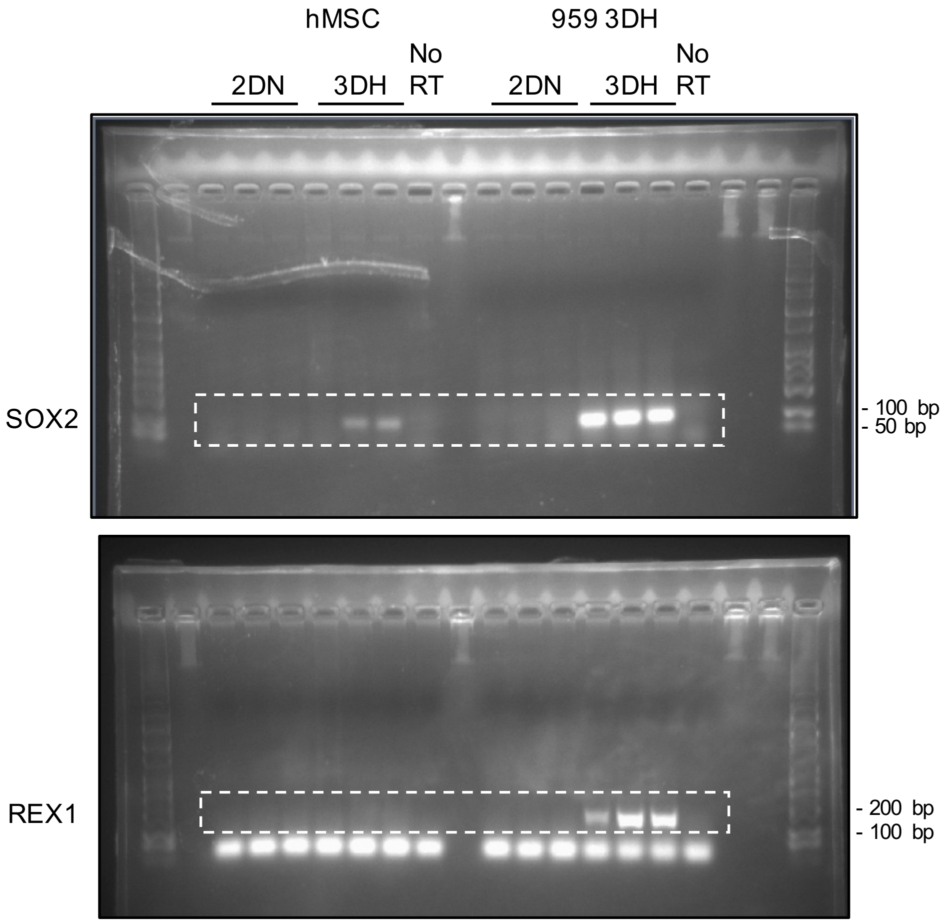

Figure S5. Full length gels of Figure 5A. White dashed lines indicate cropped areas.

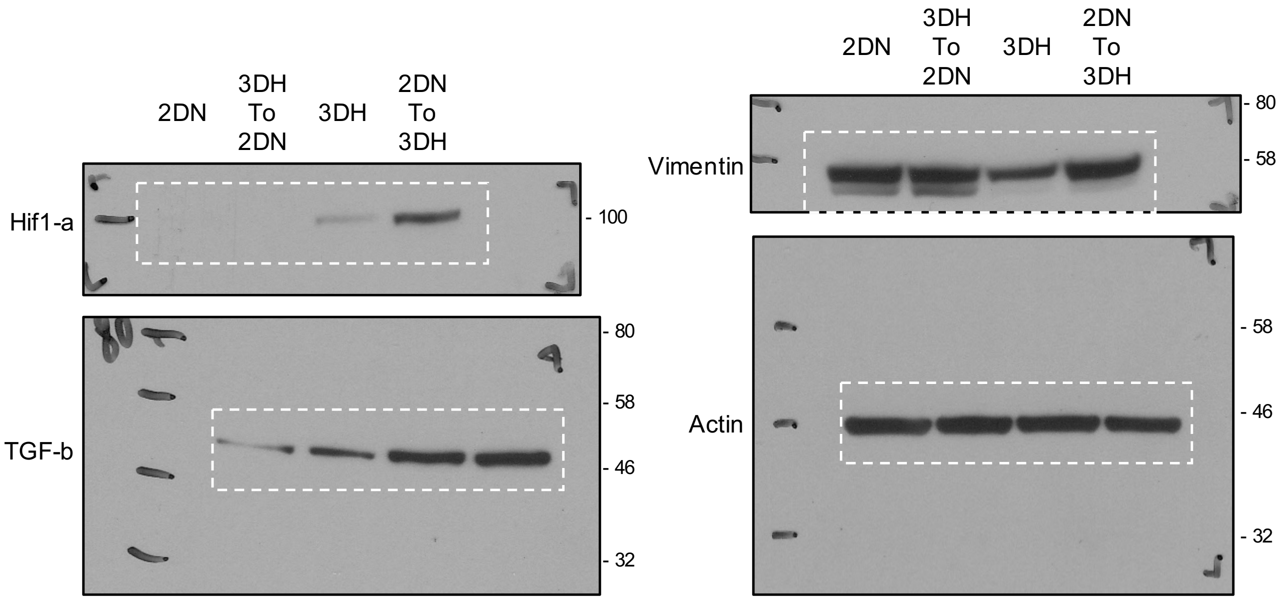

Figure S6. Full length blots of Figure 5C. White dashed lines indicate cropped areas.
